# Cecropin AD reduces viral load and inflammatory response against H9N2 avian influenza virus in chickens

**DOI:** 10.3389/fvets.2024.1369863

**Published:** 2024-03-28

**Authors:** Taiming Zhang, Zhenyi Liu, Yan Zhi, Xinping Zhao, Mengze Du, Qian Zhang, Tao Zhang, Ge Hu

**Affiliations:** College of Animal Science and Technology, Beijing University of Agriculture, Beijing, China

**Keywords:** H9N2 avian influenza virus, cecropin AD, pulmonary health, immune response modulation, viral inhibition

## Abstract

**Introduction:**

This study focuses on evaluating the therapeutic efficacy of cecropin AD, an antimicrobial peptide, against H9N2 avian influenza virus (AIV) in chickens. Given the global impact of H9N2 AIV on poultry health, identifying effective treatments is crucial.

**Methods:**

To assess the impact of cecropin AD, we conducted *in vivo* experiments involving 108 5-week-old chickens divided into control, infected, and various treatment groups based on cecropin AD dosage levels (high, medium, and low). The methodologies included hemagglutination (HA) tests for viral titers, histopathological examination and toluidine blue (TB) staining for lung pathology, real-time PCR for viral detection, and enzyme-linked immunosorbent assays for measuring serum levels of inflammatory markers.

**Results:**

The findings revealed that cecropin AD substantially reduced lung pathology and viral load, especially at higher dosages, comparing favorably with the effects seen from conventional treatments. Moreover, cecropin AD effectively modulated mast cell activity and the levels of inflammatory markers such as IL-6, TNF-α, IFN-γ, and 5-HT, indicating its potential to diminish inflammation and viral spread.

**Discussion:**

Cecropin AD presents a significant potential as an alternative treatment for H9N2 AIV in chickens, as evidenced by its ability to lessen lung damage, decrease viral presence, and adjust immune responses. This positions cecropin AD as a promising candidate for further exploration in the management of H9N2 AIV infections in poultry.

## 1 Introduction

The H9N2 subtype of avian influenza virus (AIV), a low pathogenicity avian influenza (LPAI), poses a significant threat to the global poultry industry. While not typically causing mass mortality, it adversely affects poultry productivity and can pave the way for secondary infections by opportunistic pathogens like *Escherichia coli* (1). The growing resistance to conventional antiviral drugs, coupled with their adverse side effects and potential for harmful residues, underscores the urgent need for innovative treatment approaches (2). Furthermore, the efficacy of existing H9N2 AIV vaccines is being undermined by viral antigenic variation (3), highlighting the limitations of current strategies in managing this virus.

In the pathogenesis of avian influenza, particularly the H9N2 subtype, inflammatory responses play a pivotal role. The cytokines TNF-α and IL-6, along with interferon-γ (IFN-γ), are crucial in modulating these responses. TNF-α is known for its potent proinflammatory activities, including the activation of macrophages and lymphocytes, which are essential for combating viral infections. However, its overproduction can lead to detrimental effects such as tissue injury and dysregulated immune responses (4). Interestingly, studies indicate that the production of TNF-α and IL-6 can negatively regulate each other, suggesting a complex interplay in controlling the immune response (5). In the case of avian influenza A/H7N9, for instance, elevated levels of IL-6 and other proinflammatory cytokines have been associated with severe lung injury and less effective viral clearance, highlighting their contribution to the pathogenesis (6). Similarly, IFN-γ, though commonly elevated following influenza virus infection, has been shown to suppress protective innate lymphoid cell group II (ILC2) function, potentially exacerbating the infection. This points to a nuanced role of IFN-γ in influenza pathogenesis, where its regulation might be a key target for post-infection therapy (7). Mast cells (MC) and 5-HT also significantly contribute to the inflammatory landscape and tissue damage control during infection (8). Recognizing the crucial role of inflammatory responses in H9N2 avian influenza pathogenesis, it is essential to target these processes for effective treatment.

Cecropin AD, a representative of antimicrobial peptides (AMPs), presents a novel approach. It not only exerts direct antiviral effects but also potentially modulates inflammatory responses. This dual function positions AMPs, known for their broad-spectrum antimicrobial activity, low toxicity, and reduced risk of drug resistance (9, 10), as promising candidates in the quest for innovative treatments, particularly in light of the limited efficacy of existing antiviral drugs and vaccines. Among AMPs, insect-derived peptides like cecropins and defensins are the most diverse and widely applied (11). Insects utilize unique immune responses to fend off microbial pathogens, thanks to their vast diversity and formidable defense mechanisms. Cecropin, the first insect-derived AMP, was discovered by inducing silk moth pupae with *E. coli* and *Serratia marcescens*, marking a significant milestone in AMP research (12, 13). Cecropin AD, a hybrid peptide derived from cecropin A and cecropin D (14), is at the forefront of our study due to its remarkable antimicrobial, antiviral, and anti-tumor activities without cytotoxic effects (15, 16). Its effectiveness against various pathogens, including superior antimicrobial efficacy compared to its individual components, positions it as a low-risk, promising alternative for treating poultry diseases (17). This study specifically aims to investigate the therapeutic potential of cecropin AD in treating H9N2 AIV in chickens, exploring a novel avenue in avian influenza management. We hypothesize that cecropin AD can effectively reduce the viral load and mitigate the symptoms associated with H9N2 infection. To test this hypothesis, we will examine lung tissue slices, assess lung indices, and measure viral loads, mast cell (MC) numbers and degranulation, as well as changes in major inflammatory factors. Through this research, we aspire to provide crucial theoretical data on the antiviral action of cecropin AMPs and offer insights for developing novel anti-influenza drugs, which could be pivotal in addressing the challenges posed by H9N2 AIV in the poultry industry.

## 2 Materials and methods

### 2.1 Ethics approval and consent to participate

All animal work in this study met the minimum standards of animal welfare as described in the International Guiding Principles for Biomedical Research involving Animals (at https://grants.nih.gov/grants/olaw/Guiding_Principles_2012.pdf). The handling of birds was performed in accordance with the Guidelines of the Animal Care and Use Committee and approved by the Institute of Animal Husbandry and Veterinary Medicine (Permit number: BUA2022070). All efforts were made to alleviate animal suffering and to improve their quality of life.

### 2.2 Materials and animals preparation

In this study, 108 5 weeks-old SPF White Leghorn chickens were acquired from Beijing Boehringer Ingelheim Vetsuisse Ltd. The cecropin AD (Q/DXN 063–2021), a key investigational compound, was sourced from Viteling Antibiotic-Free Breeding Technology (China). Additionally, we used oseltamivir phosphate capsules as a western medicine positive control agent. These were obtained from Yichang East Sunshine Changjiang Pharmaceutical Co., Ltd. The experimental study also involved the low pathogenicity avian influenza virus (LPAIV) H9N2 subtype, identified by the GenBank accession numbers FJ499463-FJ499470, obtained from the China Agricultural University and propagated in 11 days-old SPF embryonated chicken eggs.

### 2.3 Experimental design and drug administration

After a week of acclimatization, the chickens were allocated into six groups: three cecropin AD dosage groups (10 mg/bird/day, 20 mg/bird/day, and 40 mg/bird/day), a virus control group, a blank control group, and a positive control group treated with oseltamivir phosphate (0.39 mg/bird/day). Each group comprised 18 chickens and was housed in separate units within an SPF aviary, ensuring uninterrupted access to feed and water. The dosages of cecropin AD, oseltamivir phosphate, and the H9N2 AIV virus were carefully determined based on preliminary experiments conducted in our laboratory. These preliminary studies were critical in optimizing the therapeutic effects and safety, ensuring the virus dosage could induce significant but non-lethal illness in the chickens.

During the infection phase, we administered the H9N2 AIV virus to the experimental groups. The virus, cultivated in the allantoic cavity of embryonated eggs, was given in a dose of 0.2 mL per chicken, using nasal and ocular drops. The negative control group received the same volume of physiological saline in a similar manner. Three days post-infection, treatment commenced with the designated dosages of cecropin AD for the respective groups and oseltamivir phosphate for the pharmaceutical control group. Concurrently, the virus and negative control groups were administered the same volume of physiological saline daily.

Throughout the experiment, the physiological and behavioral parameters of the chickens, including alertness, weight, feed intake, respiration, and movement, were closely monitored to evaluate the effects of the treatments and the infection.

### 2.4 Sample collection and procedure

Three, six, and nine days post-infection, five chickens from each group were randomly selected for analysis. In strict adherence to ethical standards for animal research, these selected chickens were humanely euthanized, followed by anatomical pathology observations. Subsequently, tissue and organ samples were collected and weighed. Notably, for the purpose of this study, the lung coefficients were calculated using the weight of a single (unilateral) lung, reflective of standard practices in avian histopathological research. After the unilateral lung was weighed, the tissues were rinsed with physiological saline and preserved in a 10% polyformaldehyde solution for future histological analysis, while the remaining lung tissue samples were stored at −80°C for total RNA and protein extractions.

For the calculation of the lung coefficient (LC), we meticulously adhered to the following formula, ensuring accuracy in our measurements:


$$\mathrm{Lung}\;\mathrm{coefficient}\;\left(LC\right)=\frac{\mathrm{Unilateral}\;\mathrm{wet}\;\mathrm{lung}\;\mathrm{weight}\;\left(g\right)}{\mathrm{Body}\;\mathrm{weight}\;\left(g\right)}\times 100\%$$

### 2.5 Histological analysis and mast cell evaluation in lung tissues

Lung tissue samples from chickens were fixed in a 4% formaldehyde solution for 4 h and subsequently cut into 0.5 cm^3^ pieces, followed by a 3–4 h rinse. The tissue then underwent a dehydration process involving an hour-long water wash and graded ethanol immersions (30 min in 75% alcohol, 20 min in 80% ethanol, followed by 20 min in 95% ethanol, with the 95% ethanol step repeated four times). After drying, the samples were cleared in a xylene and ethanol mixture, embedded in wax, and sectioned into thin layers of 4 to 6 microns for histological examination. The sections were dewaxed using xylene baths and ethanol immersions, rinsed in distilled water, and stained with hematoxylin and eosin (H&E) for general histology.

For mast cell visualization, sections underwent toluidine blue staining after acidification with hydrochloric acid for 10 min and a thorough rinse. Toluidine blue solution was applied for 40 min, followed by rinsing and air-drying. Sections were then sealed on glass slides with neutral resin and labeled for analysis.

Mast cells were quantified using the Case Viewer image analysis system, with the degranulation rate calculated as the number of degranulated mast cells divided by the total mast cell count, multiplied by 100%. This process facilitated a detailed comparison of pathological changes in the lung tissues at various time points, providing insights into the inflammatory response within the lung tissues.

### 2.6 Lung tissue total RNA extraction and real-time quantitative PCR

Total RNA was extracted from 30–50 mg of chicken lung tissue using TRIzol reagent (Takara, Dalian, China), as per the guidelines provided in the manufacturer’s instructions (18). The lung tissue was initially ground into a grayish-white powder in a mortar with liquid nitrogen. The concentration and purity of the extracted RNA were determined using a Nanodrop 2000C spectrophotometer (Thermo Fisher Scientific, Waltham, MA, United States), ensuring an appropriate A260/280 absorbance ratio. cDNA synthesis was conducted using the PrimeScript RT Reagent Kit (Takara, Dalian, China). Quantitative PCR (qPCR) analyses were performed on the CFX96 Real-Time PCR Detection System (Bio-Rad, Hercules, CA, United States), with the following thermal cycling conditions: an initial denaturation at 95°C for 2 min, followed by 40 cycles of denaturation at 95°C for 10 s and annealing/extension at a specified temperature for 30 s, accompanied by fluorescence signal acquisition (as detailed in Table 1). Melt curve analysis was conducted within a temperature range of 65–95°C. Amplification efficiencies for the target genes were maintained between 95 and 105%. The qPCR reaction mix comprised 10 μL TB Green^TM^ Premix (Takara), 0.4 μL of each forward and reverse primer, 1.5 μL cDNA, and 7.7 μL DNase/RNase-Free Deionized Water (Tiangen, Beijing, China). All samples were analyzed in triplicate, with cycle threshold (Ct) values normalized against β-actin expression levels. Relative mRNA levels were quantified using the 2−ΔΔCt method (19).

**Table 1:** Primers used for quantitative real-time polymerase chain reaction (qRT-PCR).

Genes	Sequence (5′–3′)	Product length (bp)	Accession number
GAPDH	F: CCCCCATGTTTGTGATGGGT	74 bp	NM_204305.2
	R: GCACGATGCATTGCTGACAA		
IL-6	F: CTCGTCCGGAACAACCTCAA	85 bp	NM_204628.2
	R: TCAGGCATTTCTCCTCGTCG		
IFN-γ	F: CAGATGTAGCTGACGGTGGAC	155 bp	NM_205149.2
	R: TTTCTGTGTGGAAATACTGC		
TNF-α	F: GCCAGATTGTTTGAATAAGT	127 bp	NM_204267.2
	R: TTGGCCCCCAGTGTTATCAG		
5-HT	F: CCAAGAGGAGCGCTGCTAG	189 bp	XM_046920247.1
	R: AACCTTTCTATGGTTGGTAA		

### 2.7 Statistical analysis

Data were analyzed using one-way analysis of variance (ANOVA) with GraphPad Prism version 8.3 (GraphPad Software Inc., San Diego, CA) and SPSS 20 (SPSS Inc., Chicago, IL, United States). Experimental results are presented as mean ± standard deviation (SD). Tukey’s post hoc test was applied for pairwise comparisons among treatment groups. The equation ΔCt = mean value of target gene − mean value of internal reference gene was utilized, with ΔΔCt = ΔCt − mean value of control group. A *p*-value less than 0.05 was deemed statistically significant.

## 3 Results

### 3.1 Effects of H9N2 avian influenza infection and cecropin AD treatment on body weight

**Figure 1:**
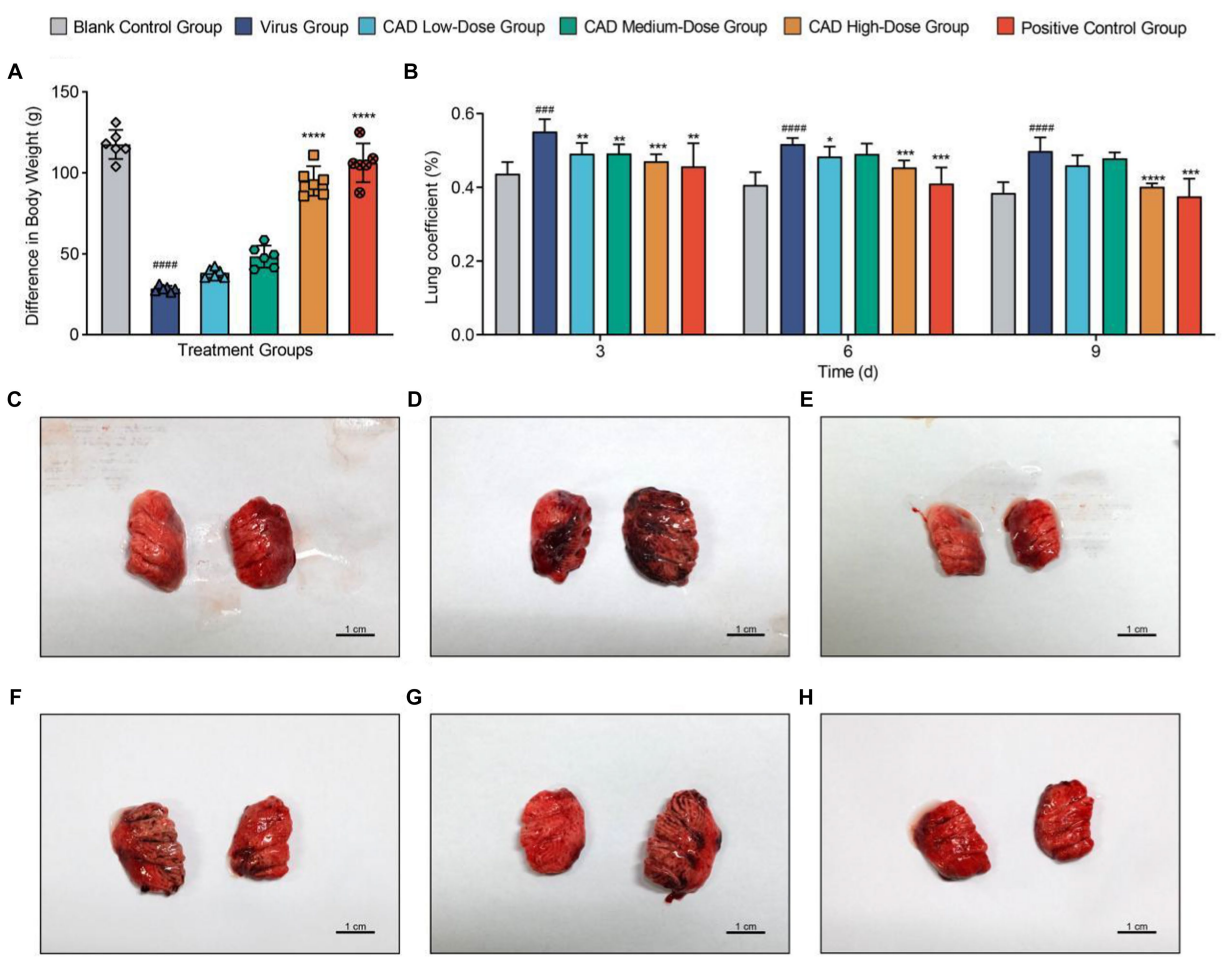
Body weight trends and lung pathology in chickens following H9N2 AIV infection and CAD treatment. **(A)** Bar graph representing body weight changes in chickens across different experimental groups, illustrating the impact of H9N2 infection and subsequent cecropin AD treatment on growth rates. **(B)** Multiple bar graph depicting lung coefficients at 3, 6, and 9 days post-infection across groups. **(C–H)** Histological examination of lung tissues from six study groups, revealing the extent of pulmonary congestion and structural changes following H9N2 infection and treatment. Congestion, mucus presence, and inflammatory infiltrates are evidenced with increasing clarity over the course of treatment, with significant reductions in pathology in the cecropin AD high-dose and positive control groups. Experiments were performed in triplicate, and the mean ± S.D. (*n* = 3 or 6) is shown. *p*-values were determined by non-parametric one-way ANOVA. Compared with virus group ^*^p < 0.05, ^**^p < 0.01, ^***^p < 0.005, and ^****^p < 0.001. Compared with the control group ^#^*p* < 0.05, ^##^*p* < 0.01, ^###^*p* < 0.005, and ^####^*p* < 0.005.

After infection with H9N2 avian influenza, the chickens exhibited symptoms including ruffled feathers, lethargy, and changes in water and feed intake. Following treatment with CAD, a gradual improvement was observed. Following virus infection, the rate and amount of weight gain in the virus group were significantly lower than those in the blank control group (*p* < 0.001). After CAD treatment, the issue of slow weight gain gradually alleviated, showing a dose-dependent effect. Significant differences were observed in the CAD high-dose group and the positive control group compared to the virus-infected group (*p* < 0.001, Figure 1A).

### 3.2 Pathological anatomy observations

Dissections revealed significant congestion and mucus in the lungs and trachea of chickens infected with H9N2 avian influenza (Figures 1C–H). Additionally, some chickens showed a reduction in thymus size, pericardial effusion, and liver congestion. The lung coefficient analysis indicated a significant increase in the virus group compared to the control group (*p* < 0.005). Treatment with cecropin AD and western medicine led to reductions in lung lesions and edema to varying degrees. After 3, 6, and 9 days of treatment, the lung coefficient in the high-dose cecropin AD group and the positive control group treated with western medicine was significantly lower than that in the virus group (*p* < 0.005, Figure 1B).

### 3.3 Pathological anatomy observations in lung tissues

**Figure 2:**
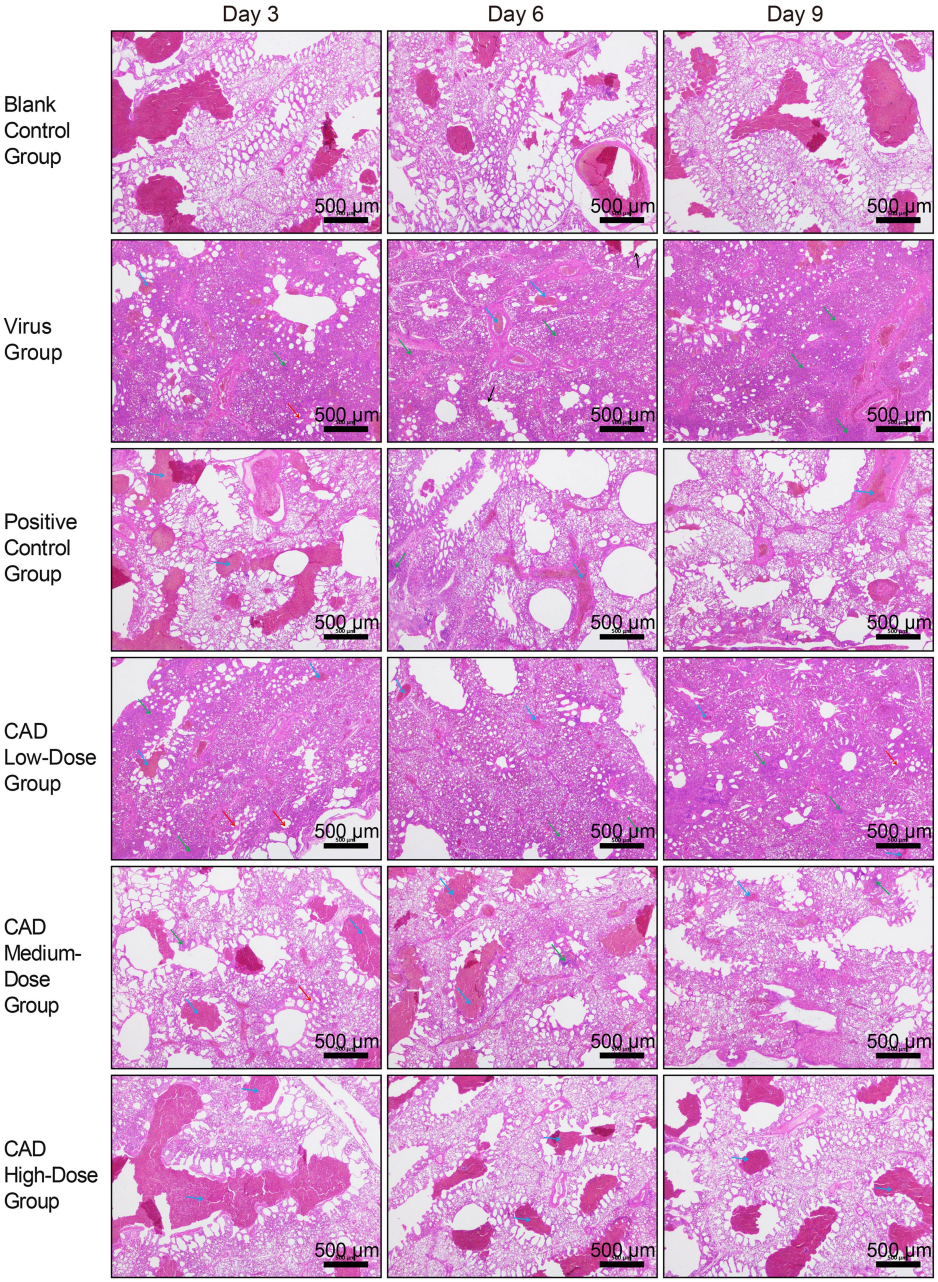
Histopathological analysis of lung tissues in chickens following H9N2 AIV infection and CAD treatment (40×). The group designations, noted on the left margin of each row, from top to bottom, include the blank control group, virus group, positive control group, and cecropin AD-treated groups at low, medium, and high doses. The time points post-infection, indicated at the top of each column, are day 3, day 6, and day 9, respectively. Scale bar = 500 μm. Annotations within the image are indicated by arrows of different colors to highlight specific histopathological features: blue arrows point to areas of congestion and stasis; green arrows indicate inflammatory cell infiltration; red arrows mark the thickening of the alveolar walls; and black arrows denote the rupture of alveolar septa.

As shown in Figure 2, 3 days post-infection, the lungs of the H9N2-infected chickens showed significant pathology, including thickened alveolar walls and patchy red blood cell clustering, indicative of congestion and stasis. Inflammatory cell infiltration was extensive in the alveolar interstitium, intensifying with increasing viral titer.

By day 6, lung tissue morphology in the virus group exhibited more severe disruption, with alveolar septa fragmentation and high red blood cell counts, reflecting worsened congestion and stasis. Inflammatory responses intensified, marked by increased neutrophils, lymphocytes, and leukocytes in the alveolar interstitium.

On day 9, lung condition improved slightly compared to day 6, with better bronchiole and alveoli integrity and reduced alveolar septal damage. However, severe inflammatory response persisted, with extensive inflammatory cell infiltration in the alveolar septa.

Throughout the 3, 6, and 9 days post-treatment period, the virus group consistently displayed lung congestion, stasis, thickened alveolar walls, and extensive inflammatory cell infiltration, albeit with some easing of symptoms by day 9. The cecropin AD and western medicine groups showed similar pathological changes but to a significantly lesser degree than the virus group. The low-dose cecropin AD group exhibited the most severe lung damage among the dosage groups, while the high-dose group demonstrated better pulmonary health.

### 3.4 Viral expression in lung tissues post-cecropin AD treatment

**Figure 3:**
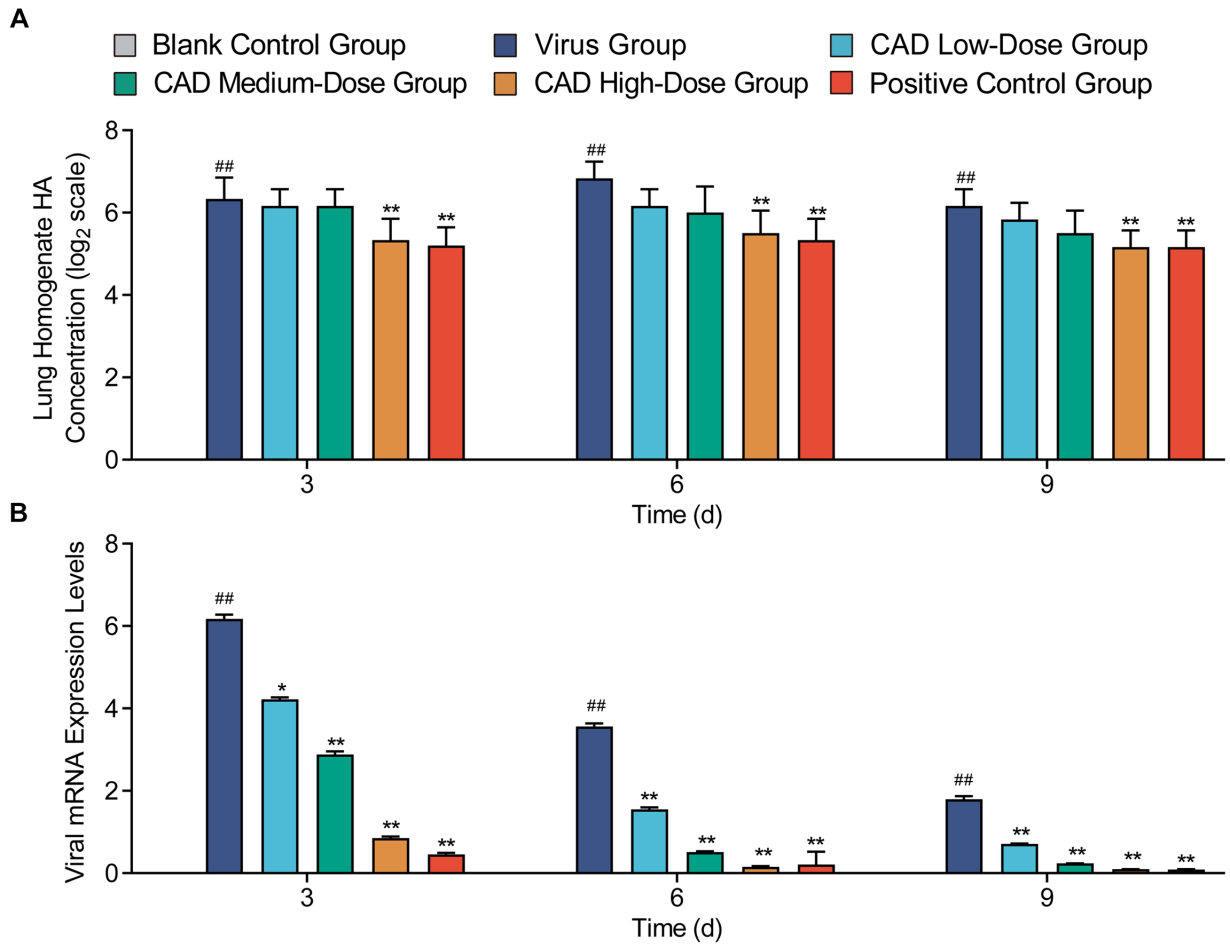
Hemagglutination titer and viral expression in lung tissues following H9N2 AIV infection and CAD treatment. **(A)** A graph showing the hemagglutination titer in lung tissue homogenates across various groups. **(B)** A graph depicting the viral expression levels in lung tissues post-treatment. Experiments were performed in triplicate, and the mean ± S.D. (*n* = 3) is shown. *p*-values were determined by non-parametric one-way ANOVA. Compared with virus group ^*^p < 0.05 and ^**^p < 0.01. Compared with the control group ^#^*p* < 0.05 and ^##^*p* < 0.01.

After 3, 6, and 9 days of treatment, the high-dose cecropin AD group exhibited a profoundly lower hemagglutination titer in lung tissue homogenates compared to the virus group (*p* < 0.01), with no significant difference observed in comparison to the western medicine group (*p* > 0.05) (Figure 3A). Cecropin AD treatment significantly reduced viral expression within the lung tissues. Following 3, 6, and 9 days of treatment, low, medium, and high dosage groups all showed viral levels significantly reduced from the virus group (*p* < 0.01). In contrast, the high-dose cecropin AD group after 3 days, as well as the medium and high doses after 6 and 9 days, demonstrated viral expression levels that were comparable to those treated with western medicine (Figure 3B), indicating the efficacy of cecropin AD in diminishing viral load in lung tissues.

### 3.5 Mast cell observations in chicken lung tissue post-cecropin AD treatment

**Figure 4:**
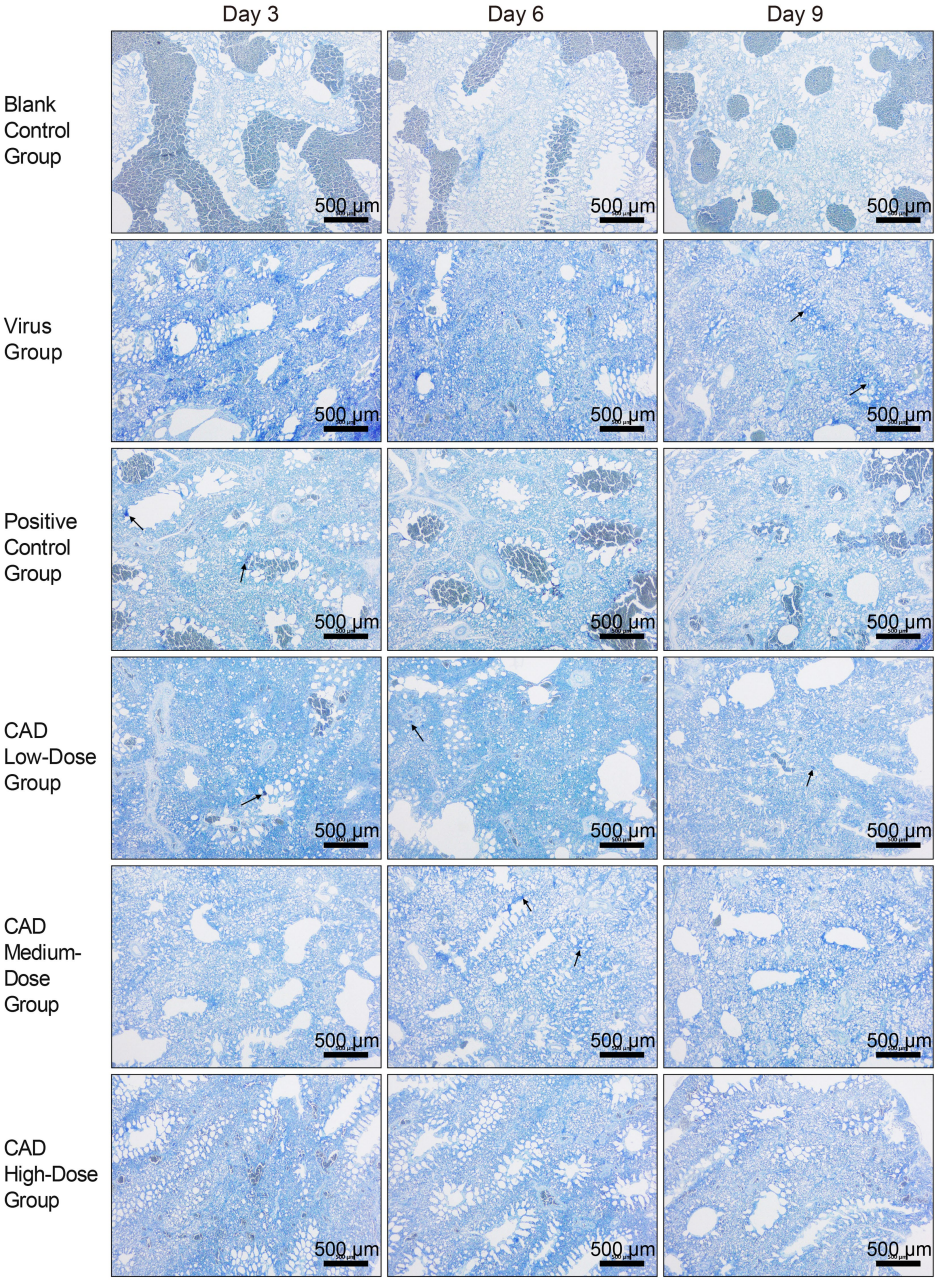
H9N2 AIV infection and CAD treatment: toluidine blue stained paraffin sections of chicken lung tissues (40×). The group designations, noted on the left margin of each row, from top to bottom, include the blank control group, virus group, positive control group, and cecropin AD-treated groups at low, medium, and high doses. The time points post-infection, indicated at the top of each column, are day 3, day 6, and day 9, respectively. Scale bar =500 μm.

After toluidine blue (TB) staining, mast cells (MCs) were distinctly identified in chicken lung tissues, with a notable concentration of MCs observed around the tracheal areas, as highlighted by black arrows in Figure 4. This observation indicates a specific distribution pattern in response to the infection, with mast cells prominently aggregating in regions likely to be affected by viral activity. The images serve as a visual representation of the variations in MC concentration and activity across different experimental groups and time points, illustrating both the spatial distribution and the physiological response of these cells to the H9N2 avian influenza virus.

Furthermore, detailed examination of these stained sections under higher magnification revealed evidence of mast cell degranulation, a process indicative of an active immune response to the infection. Degranulation, characterized by the release of granules from the mast cells, was evident in areas with high MC concentrations, particularly in proximity to the tracheal regions. This degranulation process, while not captured at the initial magnification, underscores the dynamic role of mast cells in mediating inflammatory responses within the lung tissues.

**Figure 5:**
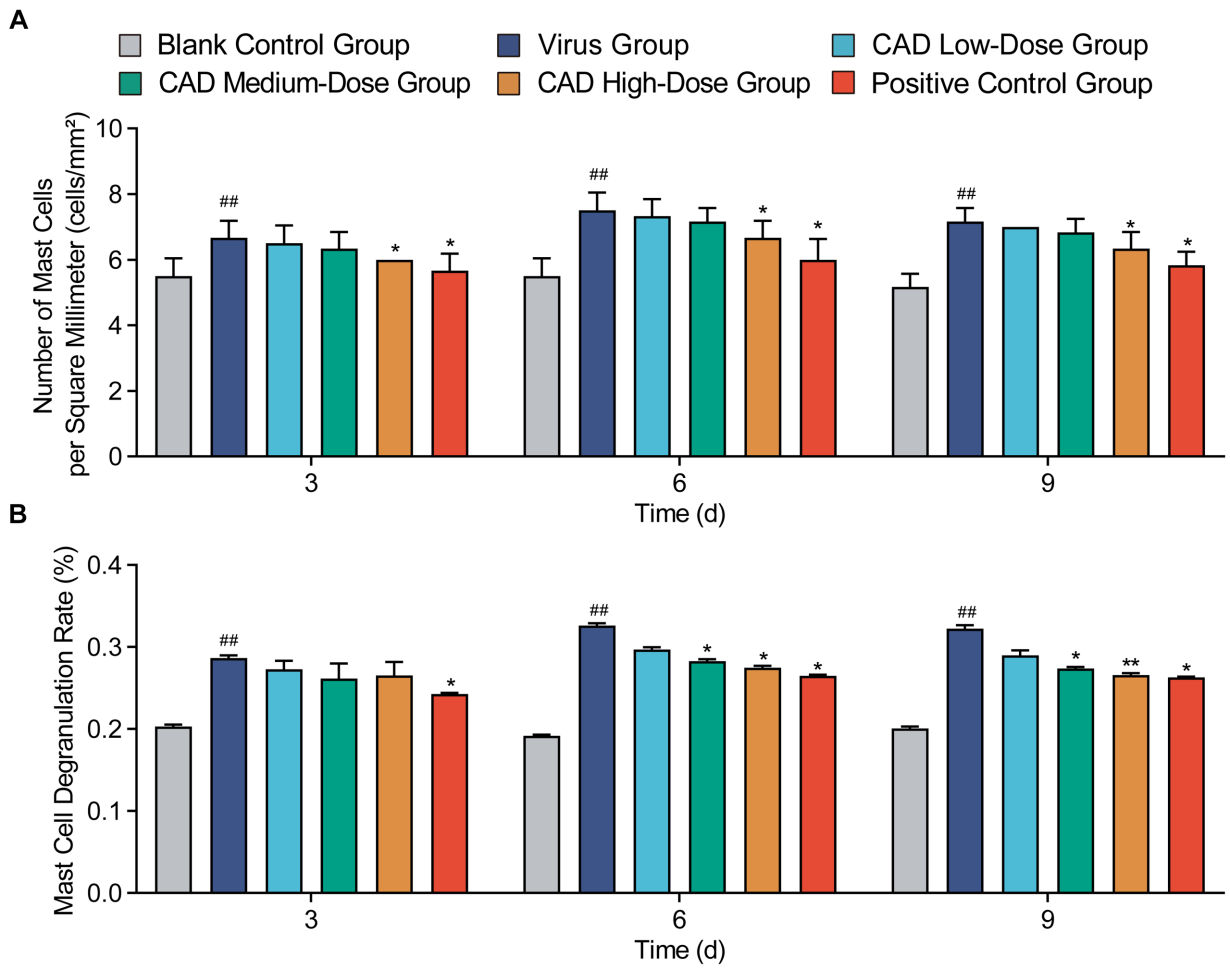
Variations in mast cell counts and degranulation rates in lung tissues following H9N2 AIV infection and CAD treatment. **(A)** The count of mast cells (MCs) in the lung tissues of chickens across different groups. **(B)** The degranulation rate of mast cells (MCs) in the lung tissues of chickens across various groups. Experiments were performed in triplicate, and the mean ± S.D. (*n* = 6) is shown. *p*-values were determined by non-parametric one-way ANOVA. Compared with virus group ^*^p < 0.05 and ^**^p < 0.01. Compared with the control group ^#^*p* < 0.05 and ^##^*p* < 0.01.

Cecropin AD treatment significantly affected the number and degranulation rate of mast cells (MCs). As shown in Figure 5, compared to the control group, there was a significant increase in the number of MCs and their degranulation rate following viral infection (*p* < 0.01). After 3, 6, and 9 days of treatment, the high-dose cecropin AD group exhibited notably lower MC counts compared to the virus group (*p* < 0.05), consistent with results observed in the western medicine group (Figure 5A). Furthermore, both medium and high doses of cecropin AD demonstrated reduced MC degranulation rates after 6 and 9 days of treatment (*p* < 0.05), with a marked decrease observed in the high-dose group by day 9 (*p* < 0.01). These changes were similar to those observed in the western medicine group (Figure 5B).

### 3.6 Cecropin AD suppression of inflammatory factor expression in chicken lungs

**Figure 6:**
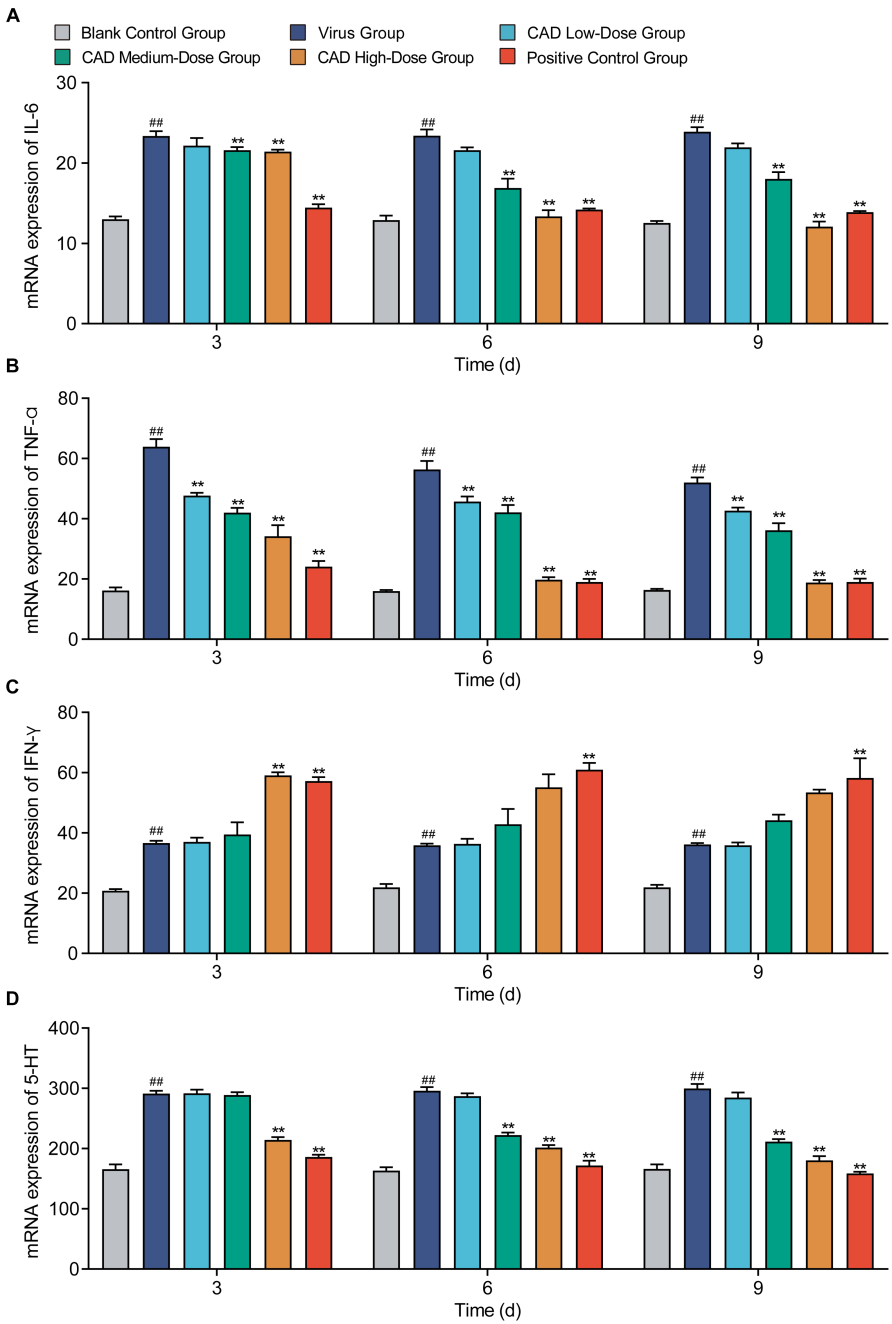
Cytokine levels changes following H9N2 AIV infection and CAD treatment. **(A–D)** Variations in levels of IL-6 **(A)**, IFN-α **(B)**, IFN-γ **(C)**, and 5-HT **(D)** across different treatment groups over time. Experiments were performed in triplicate, and the mean ± S.D. (*n* = 6) is shown. *p*-values were determined by non-parametric one-way ANOVA. Compared with virus group ^*^p < 0.05 and ^**^p < 0.01. Compared with the control group ^#^*p* < 0.05 and ^##^*p* < 0.01.

Cecropin AD treatment markedly influenced the levels of several key inflammatory factors in the chicken lungs. Both medium and high doses significantly reduced IL-6 levels after 6 and 9 days of treatment compared to the virus group (*p* < 0.01), with the medium dose showing similar efficacy to western medicine (*p* > 0.05, Figure 6A). Across all doses, cecropin AD substantially decreased TNF-α levels after 3, 6, and 9 days of treatment (*p* < 0.01), with the high dose achieving results comparable to western medicine (*p* > 0.05, Figure 6B). Notably, the high dose of cecropin AD significantly reduced IFN-γ levels after 3 days of treatment, on par with the western medicine group (*p* > 0.05, Figure 6C). Furthermore, high and medium doses of cecropin AD were effective in significantly lowering 5-HT levels after 3, 6, and 9 days (*p* < 0.01), with the high dose mirroring the efficacy of western medicine (*p* > 0.05, Figure 6D).

## 4 Discussion

Our study’s investigation into CAD efficacy against H9N2 avian influenza virus (AIV) in chickens corroborates and expands upon existing antiviral peptide research. We observed a significant reduction in lung pathology and viral load in CAD-treated groups, echoing previous findings and highlighting CAD’s potential in treating respiratory viral infections (20). The dose-dependent decrease in viral replication in lung tissues is especially relevant given H9N2 AIV’s rapid mutation rate, which poses challenges to conventional treatments. Furthermore, the modulation of key inflammatory markers, such as IL-6 and TNF-α (21), in our study aligns with the documented anti-inflammatory effects of similar peptides (22). These findings endorse CAD’s therapeutic potential in managing viral-induced inflammation and add to the evidence supporting antimicrobial peptides as alternatives to traditional antiviral therapies (23, 24), especially in the context of increasing drug resistance and safety concerns.

Our in-depth investigation of cecropin AD’s (CAD) mechanisms against H9N2 avian influenza virus (AIV) delineates its multifaceted approach. The observed significant reduction in viral load, particularly with higher doses, implicates CAD’s efficacy in either disrupting viral structures or hindering replication (25). Moreover, CAD’s role in attenuating lung pathology and diminishing inflammatory cytokines underscores its capacity for modulating immune responses (16, 26). This dual functionality, adeptly balancing antiviral and immunomodulatory actions, is essential in managing infections where excessive inflammation is detrimental. Our study further highlights the criticality of dose optimization in CAD’s therapeutic application, evidenced by the pronounced dose-dependent effects. Precise dosage is key to achieving an optimal therapeutic balance, especially in the poultry industry, where safety and efficacy of treatment are of utmost concern (27). CAD’s versatility in reducing viral replication and modulating inflammatory responses earmarks it as a potential agent for broader applications in poultry health and possibly in human therapeutics (25).

This investigation into cecropin AD’s (CAD) efficacy against H9N2 avian influenza virus (AIV) in chickens offers crucial insights for poultry health management and broader public health considerations. CAD’s notable impact in reducing viral load, alleviating lung pathology, and modulating inflammatory responses underscores its potential as an effective intervention against avian diseases. The significance of these findings becomes particularly pertinent in light of ongoing challenges in managing AIV outbreaks, which bear substantial economic and health ramifications for the poultry sector. CAD’s role in diminishing the economic impact of AIV infections and enhancing overall poultry health is commendable. Beyond avian health, the implications of this study extend to a wider context. The antiviral and anti-inflammatory properties of CAD underscore the potential of antimicrobial peptides in addressing various viral infections, particularly amidst concerns regarding drug-resistant strains and the quest for safe and effective antiviral agents. Future research avenues that warrant exploration include delving deeper into CAD’s mechanisms of action, its safety profile, and the potential for resistance development. In sum, our findings contribute to the growing body of evidence supporting the use of antimicrobial peptides like CAD in managing viral infections, offering a novel dual-action strategy against both viral replication and inflammation for broader therapeutic applications. However, our study is not without its limitations. While focusing on chickens provides valuable initial data, it restricts the findings’ applicability to other species. The controlled laboratory settings may not entirely mimic the natural environments where AIV is prevalent. The sample size, adequate for preliminary investigations, necessitates enlargement in subsequent studies to enhance robustness. Furthermore, the long-term effects and potential resistance development against CAD have not yet been addressed, which are critical aspects for understanding its enduring efficacy and practical utility. Future studies should broaden the scope to include diverse avian species and field trials, enabling a more comprehensive evaluation of CAD’s effectiveness. Investigating CAD’s role in co-infection scenarios, common in poultry settings, and elucidating its molecular mechanisms are vital for optimizing its application and developing improved formulations. Additionally, exploring CAD’s application in human medicine, especially for respiratory viruses, presents a fascinating avenue for research. Our study establishes a groundwork for further exploration into cecropin AD’s role as an antiviral agent, with the goal of fully harnessing its capabilities in managing viral infections in both veterinary and potentially human contexts.

## 5 Conclusion

Our study highlights the significant role of cecropin AD in mitigating lung damage and pulmonary edema caused by H9N2 avian influenza virus (AIV) in chickens. Cecropin AD effectively inhibits H9N2 AIV replication in the lungs and modulates key serum cytokines including IL-6, TNF-α, IFN-γ, and 5-HT. This modulation results in a notable reduction of the inflammatory response, leading to a significant antiviral effect. These findings demonstrate cecropin AD’s potential in preserving respiratory health in avian species, showcasing its efficacy as both a therapeutic and a potential preventive measure against viral respiratory infections.

## Data availability statement

The original contributions presented in the study are included in the article/supplementary material, further inquiries can be directed to the corresponding author.

## Ethics statement

The animal study was approved by the Institute of Animal Husbandry and Veterinary Medicine (Permit number: BUA2022070). The study was conducted in accordance with the local legislation and institutional requirements.

## Author contributions

TaiZ: Investigation, Visualization, Writing – original draft. ZL: Data curation, Visualization, Writing – original draft. YZ: Data curation, Formal analysis, Writing – review & editing, Visualization. XZ: Data curation, Formal analysis, Writing – review & editing. MD: Project administration, Supervision, Writing – review & editing. QZ: Project administration, Supervision, Writing – review & editing. TaoZ: Funding acquisition, Project administration, Supervision, Writing – review & editing. GH: Funding acquisition, Project administration, Supervision, Writing – review & editing.
